# Supplementary material for: Cholesterol-Induced Metabolic Reprogramming in Breast Cancer Cells Is Mediated via the ERRα Pathway
Source: Cancers (Basel). 2021 May 26;13(11):2605. doi: 10.3390/cancers13112605 (PMC8198778; doi:10.3390/cancers13112605)
Supplement: Supplementary file 1 [file cancers-13-02605-s001.zip › cancers-1162209-SI.pdf]

# Supplementary Materials: Cholesterol-Induced Metabolic Reprogramming in Breast Cancer Cells Is Mediated via the ERR$\alpha$ Pathway

**Faegheh Ghanbari, Anne-Marie Fortier, Morag Park and Anie Philip**

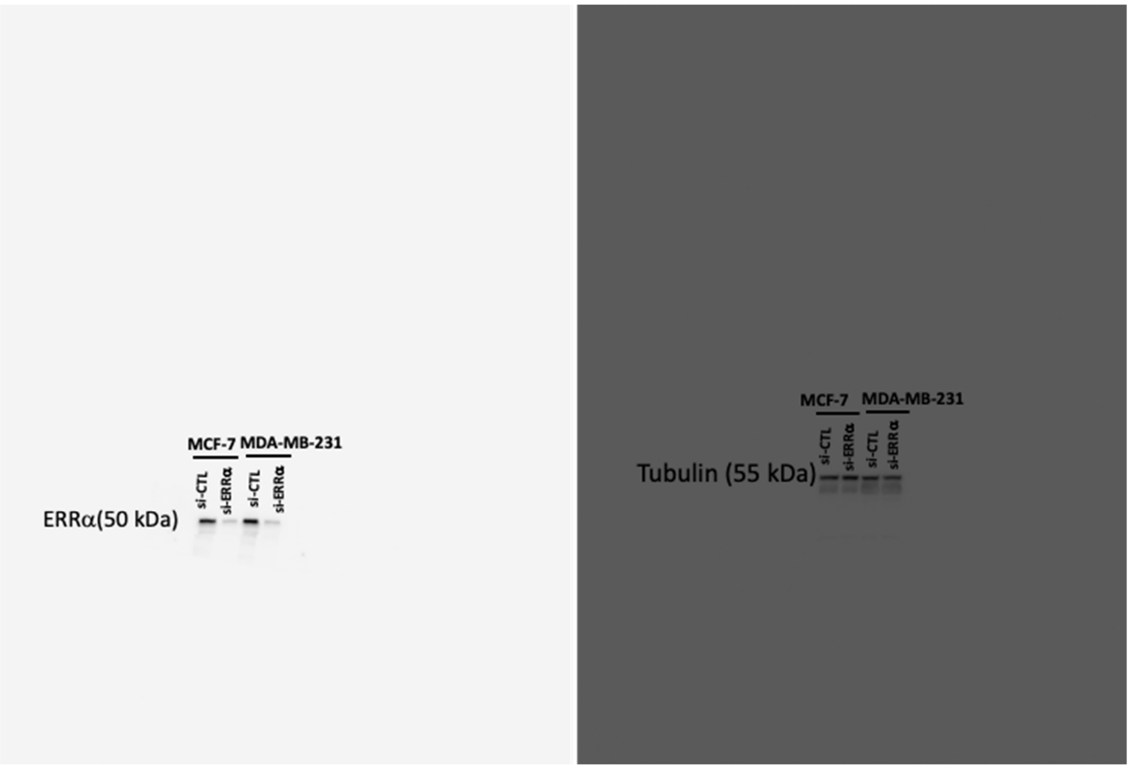


**Figure S1.** Uncorrupted western blot for Figures 1B &1G.

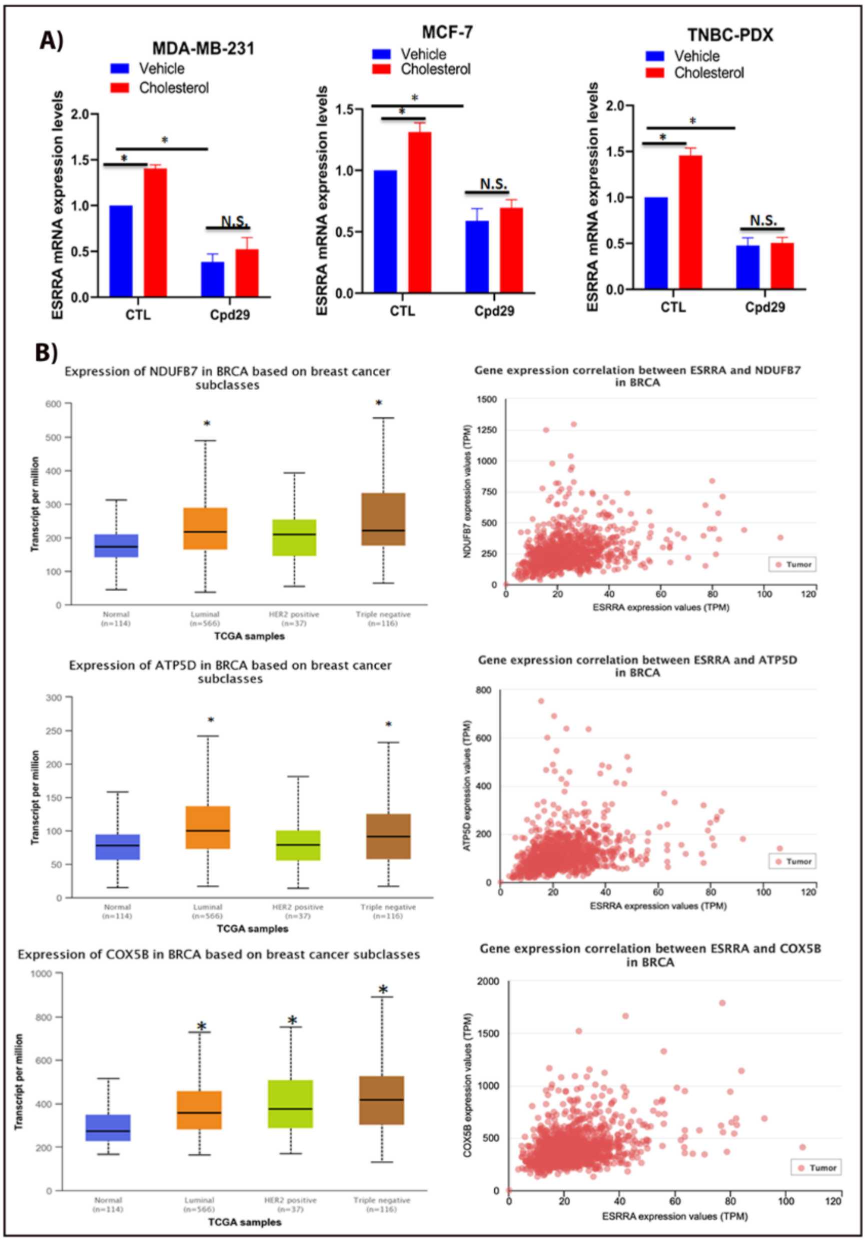


**Figure S2.** ERRα gene expression levels are positively correlated with the key enzymes involved in OXPHOS using TCGA databases. (**A**) The relative mRNA levels of ESRRA in MDA-MB-231, MCF-7, and TNBC-PDX cells. Total RNA was extracted and analyzed using RT-qPCR. MDA-MB-231 cells were treated with vehicle (Veh), cholesterol (Chol, 5 µM) and/or cpd29 (5 µM) for 48 h. MCF-7 and TNBC-PDX cells were treated with vehicle (Veh), cholesterol (Chol, 10 µM) and/or cpd29 (10 µM) for 48 h. (**B**) The data represents the mRNA levels of the indicated genes in breast tumors and corresponding normal tissue obtained from the TCGA database. The graphs were visualized using the UALCAN web-portal. Expression of NADH dehydrogenase [ubiquinone] 1 beta subcomplex subunit **7 (**NDUFB7) and ATP synthase subunit delta, mitochondrial (ATP5D) and COX5B: Cytochrome *c* oxidase subunit 5B in different subtypes of breast cancer and their correlation to the ESRRA gene expression in breast tumors. The significance (*) was defined by comparing each subtype to the normal tissue.

**Table 1.** The human primers used for qPCR.

| Name | Oligo Name | Sequence (5′ to 3′) |
|---|---|---|
| Glyceraldehyde 3-phosphate dehydrogenase | GAPDH Forward(F) | GTCTCCTCTGACTTCAACAGCG |
| | GAPDH Reverse (R) | ACCACCCTGTTGCTGTAGCCAA |
| NADH dehydrogenase [ubiquinone] 1 beta subcomplex subunit 7 | NDUFB7 F | CTGCTCAAGTGCAAGCGTGACA |
| | NDUFB7 R | CGCTCAAACTCCTTCATGCGCA |
| Cytochrome *c* oxidase subunit 5B, mitochondrial | COX5B F | GGAGATCATGCTGGCTGCAAAG |
| | COX5B R | GCAGCCTACTATTCTCTTGTTGG |
| ATP synthase subunit g, mitochondrial | ATP5L F | CCTGCTGAGATCCCTAGAGCTA |
| | ATP5L R | CCGACATAAAACCACATCAACACC |
| Methionine synthase | MTR F | CCAACTTGTCCTTCTCCTTCCG |
| | MTR R | CATACACAGGGAGGTTTCCAGC |
| Trifunctional purine biosynthetic protein adenosine-3 | GART F | GCACATCTCTGCCTGTTTGGCT |
| | GART R | CATGGAACACCTCCAGTCCTAG |
| Glucose-6-phosphate dehydrogenase | G6PD F | CTGTTCCGTGAGGACCAGATCT |
| | G6PD R | TGAAGGTGAGGATAACGCAGGC |
| 6-Phosphogluconate dehydrogenase | 6PGD F | GTTCCAAGACACCGATGGCAAAC |
| | 6PGD R | CACCGAGCAAAGACAGCTTCTC |
| Estrogen-related receptor alpha | ESRRA F | CCACTATGGTGTGGCATCCTGT |
| | ESRRA R | GGTGATCTCACACTCGTTGGAG |
| Citrate synthetase | CS F | CACAGGGTATCAGCCGAACCAA |
| | CS R | CCAATACCGCTGCCTTCTCTGT |
| Fumarate hydratase | FH F | CCGCTGAAGTAAACCAGGATTATG |
| | FH R | ATCCAGTCTGCCATACCACGAG |
| Aconitase 2 | ACO2 F | CAATCGTCACCTCCTACAACAGG |
| | ACO2 R | GTCTCTGGGTTGAACTTGAGGG |

**Table 2.** Basal-like breast tumor patient identification obtained from GEO: GSE78958 study.

| BMI status | Patient ID |
|---|---|
| BMI>25 N=16 | GSM2082151 |
| | GSM2082188 |
| | GSM2082294 |
| | GSM2082303 |
| | GSM2082370 |
| | GSM2082160 |
| | GSM2082163 |
| | GSM2082205 |
| | GSM2082300 |

| | |
|---|---|
| | GSM2082310 |
| | GSM2082440 |
| | GSM2082325 |
| | GSM2082454 |
| | GSM2082382 |
| | GSM2082340 |
| | GSM2082298 |
| BMI ≤ 25<br>N=15 | GSM2082095 |
| | GSM2082109 |
| | GSM2082169 |
| | GSM2082177 |
| | GSM2082274 |
| | GSM2082352 |
| | GSM2082384 |
| | GSM2082400 |
| | GSM2082385 |
| | GSM2082297 |
| | GSM2082234 |
| | GSM2082341 |
| | GSM2082379 |
| | GSM2082270 |
| | GSM2082423 |
